# High expression level of serpin peptidase inhibitor clade E member 2 is associated with poor prognosis in lung adenocarcinoma

**DOI:** 10.1186/s12931-020-01597-5

**Published:** 2020-12-14

**Authors:** Ryota Dokuni, Tatsuya Nagano, Naoe Jimbo, Hiroki Sato, Tatsunori Kiriu, Yuichiro Yasuda, Masatsugu Yamamoto, Motoko Tachihara, Kazuyuki Kobayashi, Yoshimasa Maniwa, Yoshihiro Nishimura

**Affiliations:** 1 Division of Respiratory Medicine, Department of Internal Medicine, Kobe University Graduate School of Medicine, 7-5-1 Kusunoki-cho, Chuo-ku, Kobe, 650-0017 Japan; 2 Department of Diagnostic Pathology, Kobe University Graduate School of Medicine, 7-5-1 Kusunoki-cho, Chuo-ku, Kobe, 650-0017 Japan; 3 Division of Thoracic Surgery, Kobe University Graduate School of Medicine, 7-5-1 Kusunoki-cho, Chuo-ku, Kobe, 650-0017 Japan

**Keywords:** Serpin peptidase inhibitor clade E member 2, Lung cancer, Adenocarcinoma

## Abstract

**Background:**

Recent studies have revealed that serpin peptidase inhibitor clade E member 2 (SERPINE2) is associated with tumorigenesis. However, SERPINE2 expression and its role in lung adenocarcinomas are still unknown.

**Methods:**

The expression levels of SERPINE2 in 74 consecutively resected lung adenocarcinomas were analyzed by using immunostaining. Inhibition of *SERPINE2* expression by small interfering RNA (siRNA) was detected by quantitative PCR. Cell number assays and cell apoptosis assays were performed to clarify the cell-autonomous function of SERPINE2 in A549 and PC9 lung cancer cells.

**Results:**

The overall survival of patients with high SERPINE2 expression was significantly worse than that of patients with low SERPINE2 expression (P = 0.0172). Multivariate analysis revealed that SERPINE2 expression was an independent factor associated with poor prognosis (P = 0.03237). The interference of *SERPINE2* decreased cell number and increased apoptosis in A549 and PC9 cells

**Conclusion:**

These results suggest that SERPINE2 can be used as a novel prognostic marker of lung adenocarcinoma.

## Background

Serine proteinase inhibitor clade E member 2 (SERPINE2), also known as protease nexin-1 (PN-1), was first identified as a neurite-promoting factor released by cultured glioma cells [1]. In addition to glioma cells, various other cells secrete SERPINE2, including endothelial cells, fibroblasts, macrophages, platelets, smooth muscle cells, chondrocytes, astrocytes, and several types of tumor cells [2–6]. SERPINE2 was proven to be a member of the SERPINE family, which has serine protease activity [1, 7]. SERPINE2 is overexpressed in a variety of adenocarcinomas, including breast cancer [3], pancreatic cancer [8], gastric cancer [9], and colorectal cancer [10], and its high expression is correlated with the degree of cancer malignancy. A previous study demonstrated that SERPINE2 is upregulated by oncogenic activation of *RAS*, *BRAF* and *MEK1* and contributes to pro-neoplastic actions of ERK signaling in intestinal epithelial cells [10]. Therefore, SERPINE2 may be a potential therapeutic target for colorectal cancer treatment [10]. In lung adenocarcinomas in particular, high expression of SERPINE2 has been previously reported [11], but the relationship to prognosis or disease progression has never been reported. In this study, we examined the expression of SERPINE2 in 74 consecutive lung adenocarcinoma cases by immunohistochemistry using an anti-SERPINE2 antibody. In addition, we silenced *SERPINE2* in two kinds of non-small cell lung cancer (NSCLC) cell lines using siRNA and performed a cell number assay and evaluation of apoptosis to clarify the cell-autonomous function of SERPINE2 in lung adenocarcinoma cell lines.

## Methods

### Patients

During the period from January 2014 to December 2014, 74 consecutive patients were treated by complete surgical resection of lung adenocarcinoma at Kobe University Hospital, Kobe, Japan. The methods of data collection and analysis were approved by the institutional review board (permission number: 160117), and written informed consent was obtained from all patients.

### Construction of the spiral array block and pathological Studies

**Fig. 1:**
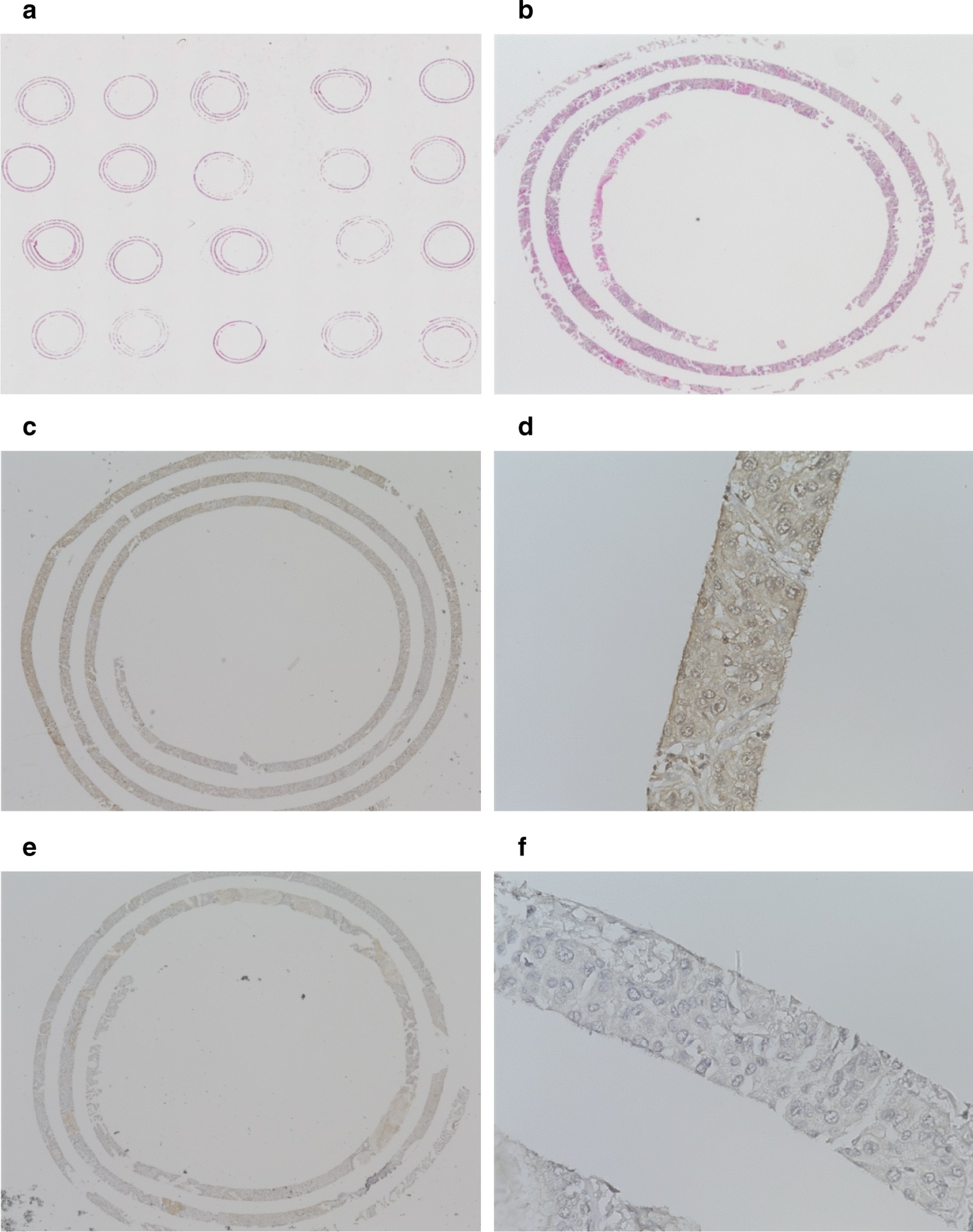
Microscopic images of spiral array specimens. Low power fields of **a** H&E staining, **c** SERPINE2 positive staining, and **e** SERPINE2 negative staining and high power fields of **b** H&E staining, **d** SERPINE2 positive staining, and **f** SERPINE2 negative staining are shown

All surgical specimens were fixed with 10% formalin and embedded in paraffin. The paraffin-embedded block was sent to the Pathology Institute (Toyama, Japan) and processed into a spiral array block. The method of preparing the spiral array block is described in detail elsewhere [12]. Briefly, 50- to 100-μm-thick slices of the sample block are cut and rolled up into cylindrical reels. These cylindrical reels are divided, and the reel containing the target site is embedded vertically in the recipient block. After that, the spiral array block is sliced and used for pathological examination. Serial 4-μm sections were stained with hematoxylin and eosin (Fig. 1a, b). All histologic specimens that had been initially evaluated by pathologists were reviewed, and the expression levels of SERPINE2 were assessed independently by two pathologists (N.J. and T.N.) who were blinded to the clinical data. The histological diagnoses were based on the 2015 WHO classification [13]. Pathological stage was determined on the basis of the TNM classification of the International Union against Cancer (UICC) [14].

### Immunohistochemical staining

Anti-human SERPINE2 antibody (Lot no. 00014298, Proteintech, Rosemont, IL, USA) was used as the primary antibody. The spiral array block was cut into 4 μm sections, which were mounted on silane-coated slides. The sections were deparaffinized in limonene (Nacalai, Kyoto, Japan) and dehydrated in a graded ethanol series. For antigen retrieval, the slides were heated for 20 min at 121 °C in 10 µM citrate buffer (pH 6.0), and endogenous peroxidase was blocked with 3% hydrogen peroxide in absolute methyl alcohol. The slides were then blocked in 2.5% horse serum for 1 h and incubated with the primary antibodies (1:200). After overnight incubation, the slides were then washed with phosphate–buffered saline and incubated with ImmPRESS Reagent (Vector laboratories, Burlingame, CA, USA). The reaction products were stained with ImmPACT DAB reagent (Vector laboratories), and the sections were counterstained with hematoxylin. A high SERPINE2 case was defined as one in which there was at least a 50% increase in positive cells compared to that in normal tissue (Fig. 1c–f).

### Cell culture

The NSCLC cell lines H460, A549 and PC9 were obtained from the American Type Culture Collection (Manassas, VA, USA). All cells were cultured in RPMI 1640 medium (Sigma, St. Louis, MO, USA) supplemented with 10% fetal bovine serum and 1% penicillin–streptomycin (Wako, Osaka, Japan) under 5% CO_2_ at 37 °C.

### SERPINE2 knockdown by small interfering RNAs (siRNAs)

The *SERPINE2* siRNAs (#1: s22188 and #2 s22189) and control siRNA (#14390843) were obtained from Thermo Fisher Scientific, MA, USA. Cells were plated in six-well plates at a density of 2 × 10^5^ cells per well. The siRNAs or control siRNA duplexes were mixed with Lipofectamine RNAiMAX Transfection Reagent (Thermo Fisher Scientific) in Opti-MEM medium (Thermo Fisher Scientific) as described by the manufacturer’s protocol and added to the plated cells. The cells were used for assessments 24 h after the addition of fresh medium.

### Quantitative reverse transcription-polymerase chain reaction (qRT-PCR)

Preparation of total cellular RNAs and qRT-PCR of the RNAs by using them as a template were performed as described previously [15]. Relative mRNA levels were calculated with the ∆∆Ct method [16] using *GAPDH* (glyceraldehyde-3-phosphate dehydrogenase) mRNA as an internal control. The primers used in this study were as follows: 5′-AATGAAACCAGGGATATGATTGAC-3′ and 5′-TTGCAAGATATGAGAAACATGGAG-3′ for *SERPINE2* and 5′-GCACCGTCAAGGCTGAGAAC-3′ and 5′-ATGGTGGTGAAGACGCCAGT-3′ for *GAPDH*.

### Effects of SERPINE2 in cell number

To determine the knockdown effects of SERPINE2 on cell growth, we used Cell Counting Kit-8 (Dojindo, Kumamoto, Japan) according to the manufacturer’s instructions. A549 and PC9 cells were plated at a concentration of 5 × 10^3^ cells/well in 96-well culture plates and analyzed 72 h later.

### Cell migration assay and cell invasion assay

To determine the knockdown effects of SERPINE2 on cell migration and invasion, we used Oris cell migration assay kit and Oris 3D invasion assay kit (Platypus technologies, WI, USA) according to the manufacturer’s instructions. A549 and PC9 cells were plated at a concentration of 2 × 10^4^ cells/well in 96-well assay plates and analyzed 0 h, 24 h, 48 h, and 72 h later. The areas of invasion were measured in ImageJ [17] and we calculated relative invasion areas compared to those at 0 h.

### Fas ligand stimulation

To investigate the involvement of SERPINE2 in the apoptosis pathway, lung cancer cell lines in which SERPINE2 was knocked down were stimulated with 200 ng/ml soluble Fas ligand (Wako, Osaka, Japan), and the temporal changes in apoptosis-related proteins were evaluated by western blotting (2, 6, 12, 24, and 48 h after Fas ligand stimulation).

### Western blotting

Primary antibodies against the following proteins were purchased from Cell Signaling Technology (Denver, MA, USA): β-actin (#4967), Bax (#2772), Bcl-2 (#4223), cleaved caspase 7 (#8438), and cleaved caspase 9 (#9505). To detect SERPINE2, we used the same antibody used for the immunohistochemical analysis at a different dilution of 1:1000. Cells were lysed in Cell Lysis Buffer (Cell Signaling Technology), and total cellular proteins (20 µg) were separated by sodium dodecyl sulfate–polyacrylamide gel electrophoresis (SDS-PAGE), which was followed by western blotting as described previously [18]. The dilution rate of primary antibody was 1:1000, and incubation performed overnight at 4 degrees Celsius. After washing in TBS-T, the membranes were treated with a HRP-conjugated anti rabbit secondary antibody for 1hour at room temperature. The blots were developed using an enhanced chemiluminescence detection kit (Thermo Fisher Scientific Inc., Waltham, MA, USA).

### Evidence from TCGA database

To evaluate the relationship between *SERPINE2* mRNA expression and the overall survival of lung adenocarcinoma patients, we utilized The Cancer Genome Atlas (TCGA) dataset through the Gene Expression Profiling Interactive Analysis (GEPIA) web server (http://gepia.cancer-pku.cn/) [19]. In lung adenocarcinoma (LUAD) dataset, we defined the patients in the 1st quartile in terms of *SERPINE2* mRNA TPM score in tumor tissue as the low SERPINE2 group and those in the 4th quartile as the high SERPINE2 group.

### Statistical analysis

All statistical analyses were performed with EZR version 1.37 (Saitama Medical Center, Jichi Medical University; http://www.jichi.ac.jp/saitama-sct/SaitamaHP.files/statmed.html; Kanda, 2018), which is a graphical user interface for R (The R Foundation for Statistical Computing, Vienna, Austria, version 3.4.1) [20]. Differences in patient characteristics between the two groups were tested for significance by the Pearson χ^2^ test or the Fisher exact test. For the univariate analysis, the cumulative survival was estimated by the Kaplan–Meier method, and differences in variables were calculated by the log-rank test. A multivariate regression analysis was conducted according to the Cox proportional hazards model. All reported P values are 2-sided, and a P value less than 0.05 was considered significant.

## Results

### Relationship between SERPINE2 expression and clinicopathologic factors

A consecutive series of 74 specimens of completely resected adenocarcinoma of the lung were examined for SERPINE2 expression. SERPINE2 expression in the cytoplasm of the cancer cells was observed in 19 of the 74 cases (26%). The relationship between SERPINE2 expression and clinicopathologic factors is shown in Table 1. SERPINE2 expression was significantly correlated with lymphatic invasion (P = 0.0188), suggesting that lung adenocarcinomas that express SERPINE2 show aggressive features. There was no relationship between SERPINE2 expression and EGFR mutation status (P = 0.416).

**Table 1:** Patient characteristics

SERPINE2	High	Low	P value
Number of case	19	55	
Age [median (range)]	69 (54–83)	72 (49–85)	
Male	13	28	0.284
Smoking history	14	30	0.181
Lobectomy	16	40	0.372
Partial resection	3	15	
pT1	10	26	0.847
pT2	9	28	
pT3	0	1	
pN0	16	43	0.121
pN1	0	8	
pN2	3	4	
pM0	19	55	
Pleural invasion	8	19	0.589
Lymphatic invasion	10	12	0.0188
Vascular invasion	9	20	0.425
pStage IA	11	25	0.163
pStage IB	5	13	
pStage IIA	0	11	
pStage IIB	0	2	
pStage IIIA	3	4	
R0	19	55	
EGFR mutation			
Ex21 L858R	5	7	0.416
Ex19 del	1	4	
Negative	9	23	
Unknown	12	13	
Adjuvant chemotherapy	4	19	0.391

Fisher’s exact test

### Relationship between SERPINE2 expression in immunostaining and overall survival

**Fig. 2:**
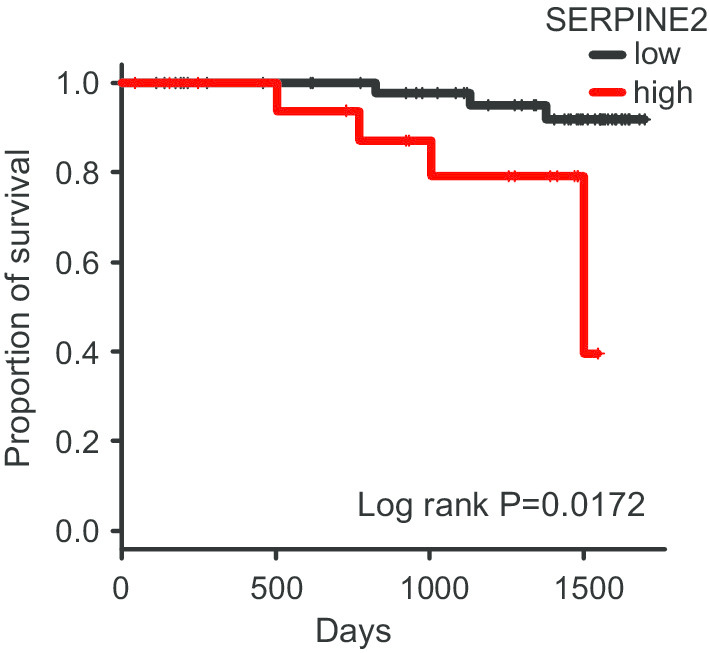
Kaplan–Meier survival curve. High SERPINE2 expression is correlated with poor prognosis

The overall survival curve obtained by the Kaplan–Meier method is shown in Fig. 2. SERPINE2 expression was significantly correlated with a short survival time (P = 0.0172). Univariate analyses showed that high expression of SERPINE2 (P = 0.03228) was correlated with a short survival time (Table 2). Table 3 shows the impact of potential prognostic factors on the survival of patients with adenocarcinoma with expression of SERPINE2 based on the results of the multivariate analysis with the Cox proportional hazards model. All significant univariate parameters were included in the multivariate analysis simultaneously. Together, these results proved that SERPINE2 was an independent predictor of a poor prognostic outcome.

**Table 2:** Univariate analysis

Variables	Cut off	Hazard ratio	95% CI for HR	P value
pT factor	< 2 vs. ≥ 2	3.08	0.596–15.91	0.1795
pN factor	0 vs. ≥ 1	2.787	0.6227–12.48	0.1801
Lymphatic invasion	0 vs. 1	2.375	0.5291–10.66	0.93750
Pleural invasion	0 vs. ≥ 1	3.383	0.7553–15.15	0.1111
Vascular invasion	0 vs. 1	2.623	0.5863–11.73	0.2072
SERPINE2	Low vs. high	5.273	1.151–24.16	0.03228

**Table 3:** Multivariate analysis

Variables	Cut off	Hazard ratio	95% CI for HR	P value
pT factor	< 2 vs. ≥ 2	2.2610	0.13140–38.900	0.57420
pN factor	0 vs. ≥ 1	3.5320	0.34880–35.770	0.28540
Lymphatic invasion	0 vs. 1	0.4981	0.06594–3.763	0.49940
Pleural invasion	0 vs. ≥ 1	0.6023	0.04930–7.358	0.69130
Vascular invasion	0 vs. 1	2.8550	0.31770–25.650	0.34910
SERPINE2	Low vs. high	9.0520	1.20300–68.090	0.03237

### Survival analysis of TCGA dataset

**Fig. 3:**
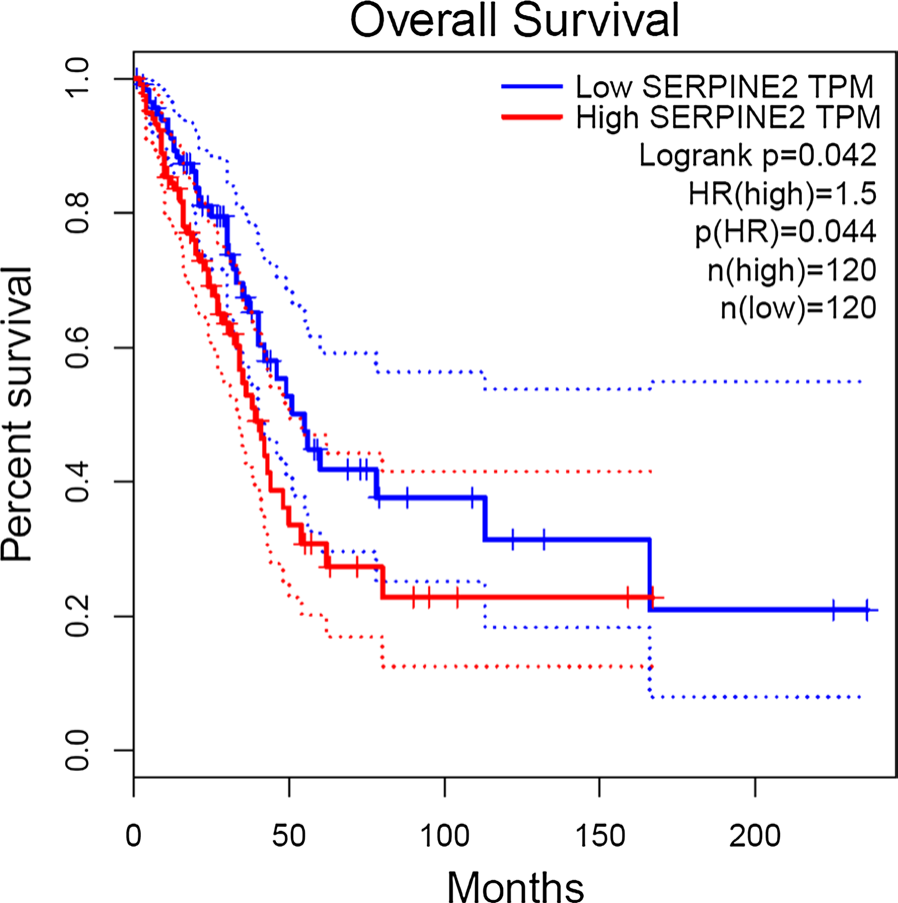
Survival analysis in TCGA dataset. The survival analysis based on TCGA dataset (LUAD) is shown

The survival analysis of the TCGA dataset performed through the GEPIA webserver also showed that the high *SERPINE2* group had a worse prognosis (P = 0.042) than the low expression group (Fig. 3).

### Effect of SERPINE2 siRNA on the autonomous behavior of cancer cells

**Fig. 4:**
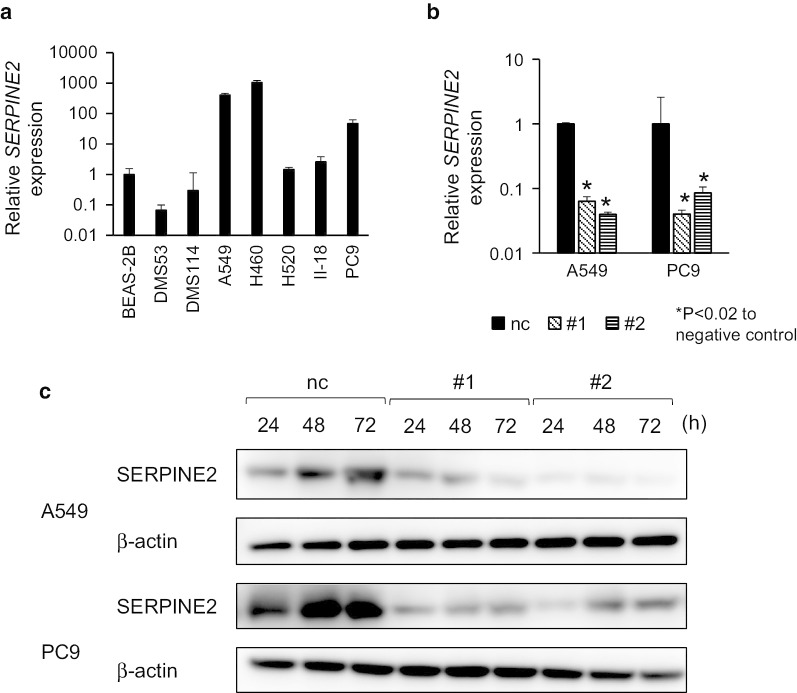
*SERPINE2* expression and knockdown effect of *SERPINE2*. **a** *SERPINE*2 expression in lung cancer cell lines was normalized to BEAS-2B. **b**, **c** The efficacies of siRNA in A549 and PC9 cells were analyzed by qRT-PCR (B, relative to negative control) and western blotting (**c**). nc, negative control; #1, siRNA#1; #2, siRNA#2

**Fig. 5:**
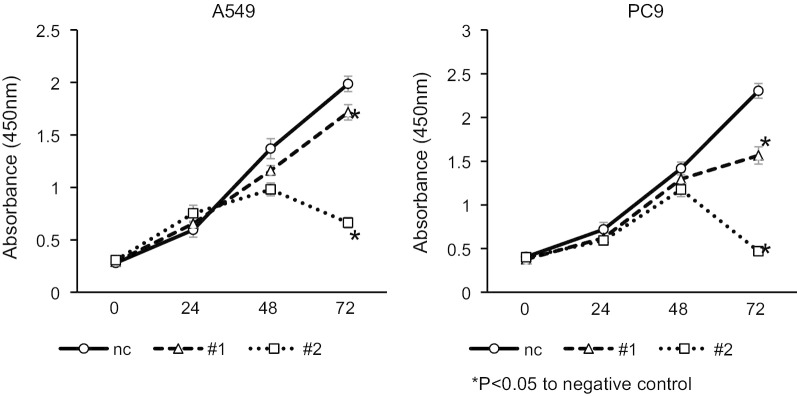
Effect of *SERPINE2* knockdown on cell number. The results of the cell number assay are shown. nc, negative control; #1, siRNA#1; #2, siRNA#2

Among lung cancer cell lines, H460, A549 and PC9 more highly express SERPINE2 compared to BEAS-2B, which is a bronchial epithelial cell line (Fig. 4a). To test the effect of increased SERPINE2 on cell autonomous behavior in vitro, the cell number of A549 and PC9 cells treated with *SERPINE2* siRNA or a negative control was examined by using a CCK-8 assay. After transfection, the interference efficiency of *SERPINE2* was first confirmed by qRT-PCR. Normalized to the expression of the negative control, *SERPINE2* expression was significantly downregulated in A549 and PC9 cells by both *SERPINE2* siRNA#1 and #2 (p < 0.05) (Fig. 4b). The protein levels of SERPINE2 were also suppressed by gene knockdown (Fig. 4c). Then, the cell number was quantified at 24, 48, and 72 h. The cell number was significantly decreased in cancer cells with *SERPINE2* downregulation (P < 0.05) (Fig. 5). This result suggests that *SERPINE2* possibly plays a role in the proliferation of cancer cells.

### The effect of SERPINE2 knockdown on Fas ligand-mediated apoptosis

**Fig. 6:**
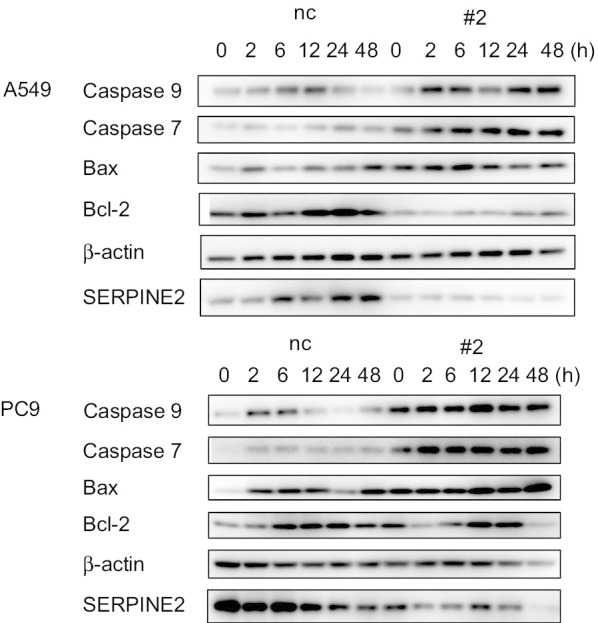
Effect of *SERPINE2* knockdown on apoptosis. Western blotting analysis of apoptosis-related proteins at the indicated time points after Fas ligand stimulation is shown. A549 (upper) and PC9 (lower) cells pretreated with SERPINE2 siRNA were stimulated with Fas ligand (200 ng/ml). nc, negative control; #2, siRNA#2

First, we performed cell migration and invasion assays, but we did not observe remarkable changes (Additional file 1: Figs. S1 and S2). Furthermore, *BMP4,* which plays a crucial role in migration and invasion, was not activated by *SERPINE2* knockdown in A549 and PC9 lung cancer cells (Additional file 1: Fig. S3). Then, to investigate the effect of SERPINE2 on apoptosis, A549 and PC9 cells treated with SERPINE2 siRNA or a negative control siRNA were stimulated with soluble Fas ligand (200 ng/ml). In both cell lines, Fas ligand-mediated apoptosis was more highly induced in cells with *SERPINE2* interference. Especially in A549 cells, the protein expression of bcl-2, an anti-apoptotic protein, was decreased (Fig. 6). Together, these results suggest that *SERPINE2* is not associated with cell migration and invasion but is associated with anti-apoptosis.

## Discussion

The current study demonstrated the role of SERPINE2 as an independent prognostic factor of lung adenocarcinomas and its molecular mechanism for the first time. In terms of prognostic factors, evaluation of the pathological specimens showed that increased expression of SERPINE2 was related to lymphatic invasion and poor overall survival. Using the GEPIA database, we found that high SERPINE2 mRNA expression was also related to poor prognosis. The overall results for survival showed the same trend as that observed in previous reports on different cancers, including breast cancers [3], gastric cancers [9] and osteosarcomas [21]. Regarding the molecular mechanism, in vitro analysis revealed that SERPINE2 plays a possible anti-apoptotic role. It may be related to the malignant features in cancer cells with high SERPINE2 expression.

Our experiment showed that silencing of *SERPINE2* resulted the decrease in cell number. Epidermal growth factor (EGF) induced SERPINE2 expression through the EGF/MEK/ERK pathway, and SERPINE2 knockdown reduced cell proliferation induced by EGF [22]. SERPINE2 is thought to act as an effector of the EGF pathway to cause cell proliferation. Furthermore, SERPINE2 is reported to inhibit plasminogen-induced apoptosis of Chinese hamster ovary fibroblasts (CHO-K1), which constitutively express tissue-type plasminogen activator (t-PA) [23]. In a previous study, transfection of the *SERPINE2* gene significantly inhibited the activity of plasmin and t-PA via the formation of inhibitory complexes and prevented cell detachment and apoptosis [23]. On the other hand, it was reported in a study using prostate cancer cells that apoptosis was induced by SERPINE2 [5], and it is believed that the effect differs depending on the cancer type. The results of the current experiment suggest that SERPINE2 suppresses apoptosis in lung cancer cell lines and may be a target molecule for lung cancer treatment.

In esophageal squamous cell carcinomas, SERPINE2 inhibition resulted in a reduction in cell growth, migration and invasion [24]. In esophageal squamous cell carcinomas, SERPINE2 promotes tumor metastasis by activating bone morphogenetic protein 4 (BMP4) [24]. Hence, we revealed no remarkable changes in cell migration and invasion assays (Additional file 1: Figs. S1 and S2) because the current study revealed that SERPINE2 was not associated with BMP4 in lung cancer cells (Additional file 1: Fig. S3).

The limitation of the current study is that a relatively small sample size is included in the current study. Hence, other prognostic factors, such as T and N factors, were not significant factors associated with poor prognosis.

## Conclusions

SERPINE2 can be a prognostic factor and might be a possible target of NSCLC by suppressing apoptosis.

## Supplementary Information

**Additional file 1:** Additional figures.

## Abbreviations

BMP4: Bone morphogenetic protein 4
CCK-8: Cell Counting Kit-8
NSCLC: Non-small cell lung cancer
qRT-PCR: Quantitative reverse transcription-polymerase chain reaction
PN-1: Protease nexin-1
SDS-PAGE: Sodium dodecyl sulfate-polyacrylamide gel electrophoresis
SERPINE2: Serine proteinase inhibitor clade E member 2
siRNA: Small interfering RNA
UICC: International Union against Cancer
