# Supplementary material for: High expression level of serpin peptidase inhibitor clade E member 2 is associated with poor prognosis in lung adenocarcinoma
Source: Respir Res. 2020 Dec 14;21:331. doi: 10.1186/s12931-020-01597-5 (PMC7737331; doi:10.1186/s12931-020-01597-5)
Supplement: Supplementary file 1 — Additional file 1: Additional figures. [file 12931_2020_1597_MOESM1_ESM.pptx]

## Slide 1
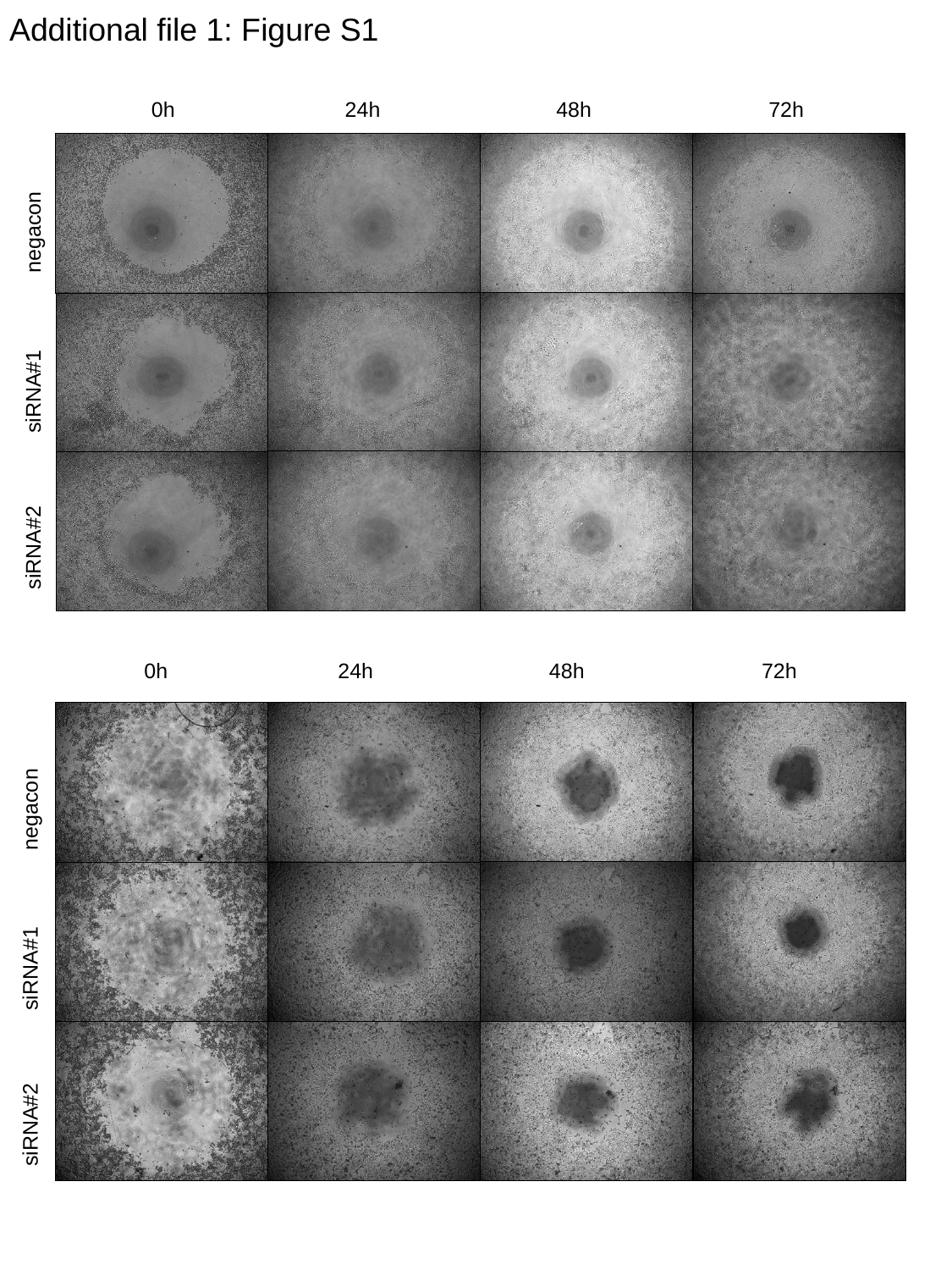

A549
Additional file 1: Figure S1
0h
24h
48h
72h
negacon
siRNA#1
siRNA#2
0h
24h
48h
72h
negacon
siRNA#1
siRNA#2

## Slide 2
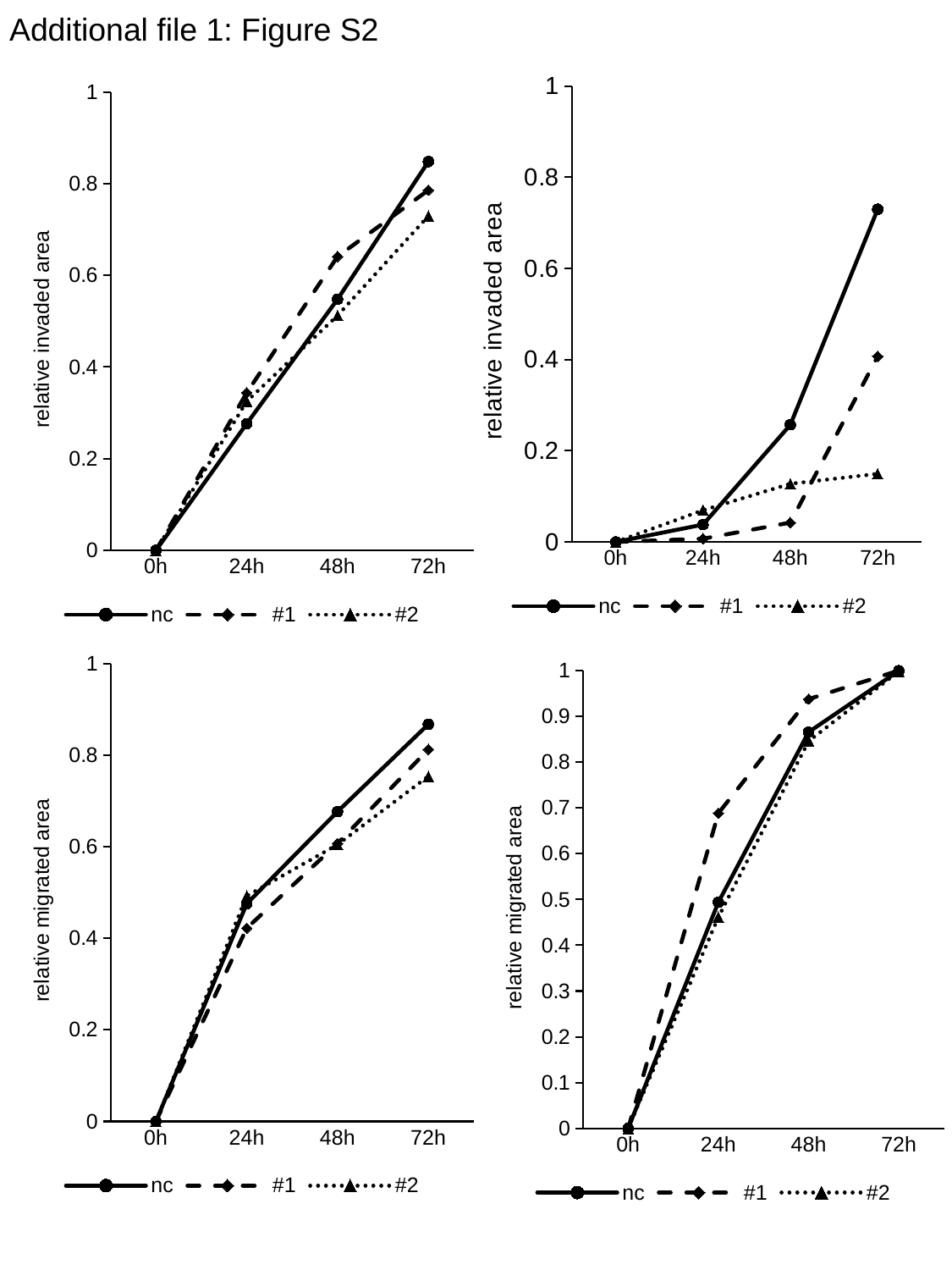

Additional file 1: Figure S2
### Chart
| Category | nc | #1 | #2 |
|---|---|---|---|
| 0h | 0.0 | 0.0 | 0.0 |
| 24h | 0.0379959488758673 | 0.007110623013604367 | 0.06946204228295573 |
| 48h | 0.25688373397721515 | 0.042344003322461195 | 0.1276016535038482 |
| 72h | 0.7298579006994156 | 0.4071405112628307 | 0.1492705194444262 |
### Chart
| Category | nc | #1 | #2 |
|---|---|---|---|
| 0h | 0.0 | 0.0 | 0.0 |
| 24h | 0.27643486451465177 | 0.34384816194739815 | 0.32495670216231975 |
| 48h | 0.5478080012767073 | 0.6409945653273733 | 0.512372035598539 |
| 72h | 0.8479469472647069 | 0.7858194843244435 | 0.7293649364679253 |
### Chart
| Category | nc | #1 | #2 |
|---|---|---|---|
| 0h | 0.0 | 0.0 | 0.0 |
| 24h | 0.4753540480355791 | 0.4218929779737466 | 0.49243619575027164 |
| 48h | 0.6765968673540858 | 0.6069029191363877 | 0.6045042085940042 |
| 72h | 0.8673556561313363 | 0.8124600056984581 | 0.7538647847201331 |
### Chart
| Category | nc | #1 | #2 |
|---|---|---|---|
| 0h | 0.0 | 0.0 | 0.0 |
| 24h | 0.493957835886087 | 0.6883074498841261 | 0.4614675531002464 |
| 48h | 0.8654963310232369 | 0.9376341285722292 | 0.8457179134076018 |
| 72h | 0.9994758604624076 | 1.0 | 0.9983375362511683 |

## Slide 3
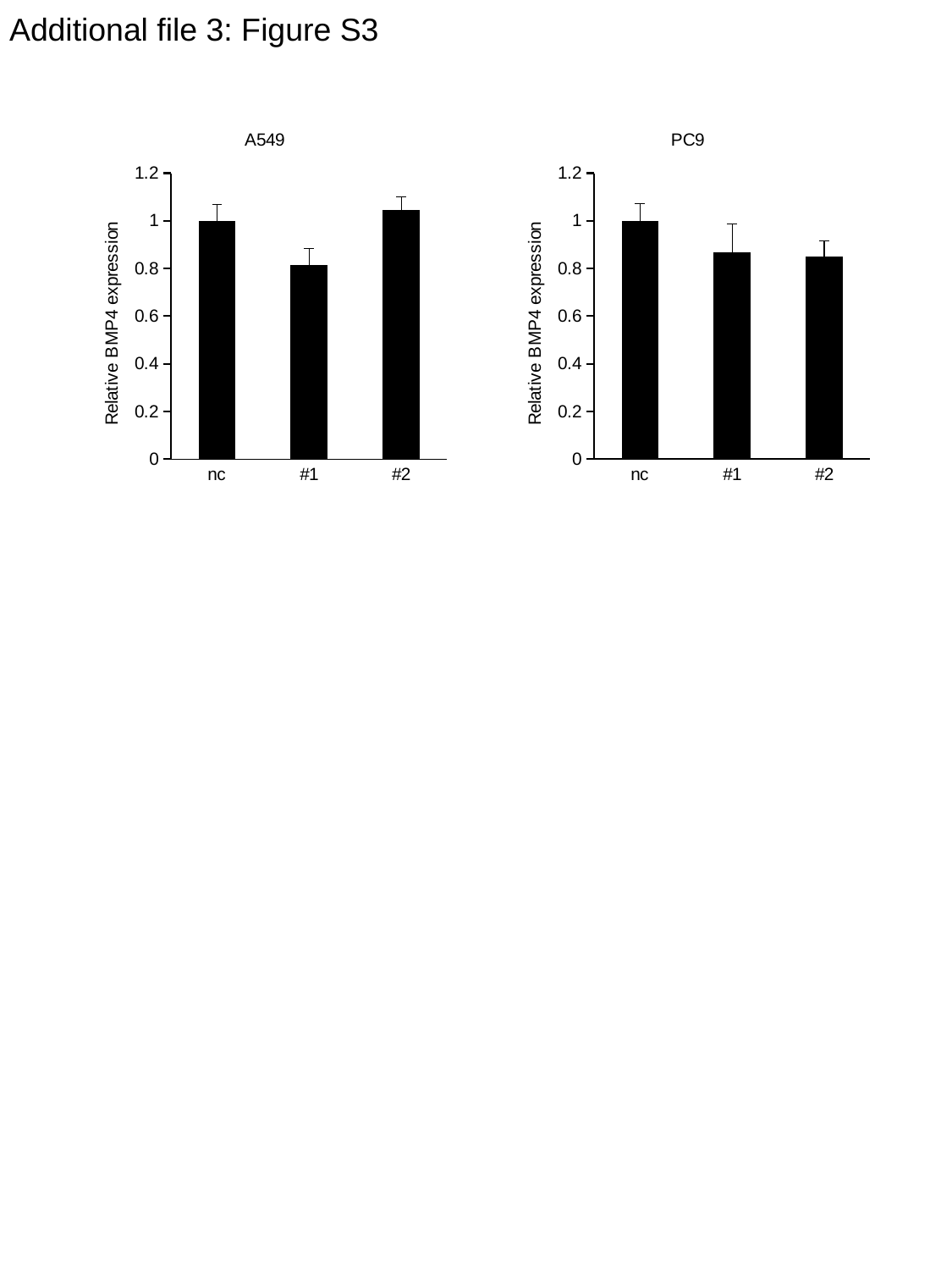

Additional file 3: Figure S3
### Chart: A549
| Category | |
|---|---|
| nc | 1.0 |
| #1 | 0.8141309377153397 |
| #2 | 1.0472933941005098 |
### Chart: PC9
| Category | |
|---|---|
| nc | 1.0 |
| #1 | 0.8685399075139193 |
| #2 | 0.8506681972666423 |
